# How Many Muscles? Optimal Muscles Set Search for Optimizing Myocontrol Performance

**DOI:** 10.3389/fncom.2021.668579

**Published:** 2021-10-07

**Authors:** Cristian Camardella, Melisa Junata, King Chun Tse, Antonio Frisoli, Raymond Kai-Yu Tong

**Affiliations:** ^1^Perceptual Robotics (PERCRO) Laboratory, TECIP Institute, Scuola Superiore Sant'Anna, Pisa, Italy; ^2^Biomedical Engineering (BME) Laboratory, Department of Biomedical Engineering, The Chinese University of Hong Kong, Hong Kong, Hong Kong, SAR China

**Keywords:** myocontrol, synergies, muscles, optimization, rehabilitation, EMG, robotics, electrodes

## Abstract

In myo-control, for computational and setup constraints, the measurement of a high number of muscles is not always possible: the choice of the muscle set to use in a myo-control strategy depends on the desired application scope and a search for a reduced muscle set, tailored to the application, has never been performed. The identification of such set would involve finding the minimum set of muscles whose difference in terms of intention detection performance is not statistically significant when compared to the original set. Also, given the intrinsic sensitivity of muscle synergies to variations of EMG signals matrix, the reduced set should not alter synergies that come from the initial input, since they provide physiological information on motor coordination. The advantages of such reduced set, in a rehabilitation context, would be the reduction of the inputs processing time, the reduction of the setup bulk and a higher sensitivity to synergy changes after training, which can eventually lead to modifications of the ongoing therapy. In this work, the existence of a minimum muscle set, called optimal set, for an upper-limb myoelectric application, that preserves performance of motor activity prediction and the physiological meaning of synergies, has been investigated. Analyzing isometric contractions during planar reaching tasks, two types of optimal muscle sets were examined: a subject-specific one and a global one. The former relies on the subject-specific movement strategy, the latter is composed by the most recurrent muscles among subjects specific optimal sets and shared by all the subjects. Results confirmed that the muscle set can be reduced to achieve comparable hand force estimation performances. Moreover, two types of muscle synergies namely “*Pose-Shared”* (extracted from a single multi-arm-poses dataset) and “*Pose-Related”* (clustering pose-specific synergies), extracted from the global optimal muscle set, have shown a significant similarity with full-set related ones meaning a high consistency of the motor primitives. Pearson correlation coefficients assessed the similarity of each synergy. The discovering of dominant muscles by means of the optimization of both muscle set size and force estimation error may reveal a clue on the link between synergistic patterns and the force task.

## 1. Introduction

Myoelectric control or myo-control is an advanced human-machine interface technique to control robots and devices in rehabilitative and assistive applications. Myo-control decodes human motor intention in the form of analyzed electromyographic (EMG) signals into a computed control signal that drives robots or machines. The rise of myo-control was initially started by the need to drive prosthetic devices, reproducing a set of distinct muscle activity patterns after performing certain contractions of the residual limb (Lowery et al., 2003; Hargrove et al., 2007). Facing the challenge of controlling multiple degrees of freedom (DoFs), the application of pattern recognition of spatio-temporal patterns of muscle activities for prostheses control significantly increased user performance in 3D movements making it more comfortable and intuitive than direct control (Hargrove et al., 2017). As a result, the classification of movements associated to daily activities reached high performance (Sensinger et al., 2009; Young et al., 2011; Adewuyi et al., 2015). Nevertheless, the intrinsic on-off and sequential nature of this control strategy determined a gradual growing interest toward simultaneous and proportional control (SPC) or simply proportional control. In the context of myoelectrical controls, a proportional control theoretically allows for a continuous support of limb or hand movements, continuously producing a control signal to an external device (e.g., robotic device) based on user's residual muscular activity. Fougner et al. defined the proportional control as a strategy with which “the user can control at least one mechanical output variable of the prosthesis within a finite and essentially continuous interval by varying his/her control input within a corresponding continuous interval” (Fougner et al., 2012). The “essentially continuous” term refers to a digital sampling interval, small enough to not affect human perception thus being negligible. The SPC paradigm opened new possibilities to design prostheses control strategies following the way human neuromuscular system activates DoFs simultaneously and proportionally (Battye et al., 1955; Bottomley, 1965; Jiang et al., 2013). In the last decade several studies have been conducted on the proportional control of robotic devices and prostheses, using linear or non-linear regression algorithms, within isometric or dynamic setups (Cheung et al., 2012; Jiang et al., 2013; Berger and d'Avella, 2014; Roh et al., 2015; Buongiorno et al., 2018). Here, muscle activations have been used for continuously estimating either articulation torques or hand force during planar reaching tasks. Also, following the trail of bio-inspired strategy development, dimensionality-reduction algorithms have been used to extract motor primitives, aiming at explaining how the human brain produces low-dimensional perceptual representations of a high number of sensory information distributed in the whole body (Hayward, 2011; Beckerle et al., 2017). These primitives, called muscle synergies or synergies, have been theorized as a way to explain motor control and learning by the central nervous system (CNS), given the abundant number of motor units in human beings and animals (d'Avella et al., 2003; Bizzi and Cheung, 2013; d'Avella, 2016). Muscle synergies have applications in a variety of fields, for example clinical assessments (Cheung et al., 2012; Roh et al., 2015) and control in robotics (Jiang et al., 2009; Berger and d'Avella, 2014): in the latter context, synergy-based myo-control was designed to exploit muscle activation patterns during task-related movements, reflecting the concept of CNS motor control. Synergy-based myo-controls have been tested in the hand force or articulation torques prediction, with linear-regression models, in a fixed (Berger and d'Avella, 2014) or multiple configurations of the limb (Buongiorno et al., 2017; Camardella et al., 2020a) with different synergies extraction algorithms. Most of the experiments in the literature exploited either isometric (in a virtual environment) or dynamic reaching tasks. In a work by Lunardini et al., synergy-based torque estimation algorithms revealed to be less sensitive to signal noise and no differences were found between isometric and dynamic protocols (Lunardini et al., 2015). They, thus, suggested that synergy-based estimations perform better than muscles-pair in a dynamic protocol in which signals are more likely to be corrupted by artifacts. In another work, Roh et al. found that synergy composition was conserved across isometric tasks with different bio-mechanical constraints (Roh et al., 2012). Similarly Muceli et al. found that synergies extracted from dynamic tasks were robust against electrode shifts, being suitable for an intensive clinical usage (Muceli et al., 2013). In both cases, synergies were useful to identify muscle activation patterns when extracted from reaching-movements EMG.

In both robotics and clinical assessment contexts, synergies extracted with the non-negative matrix factorization have always shown a physiological meaning, giving important insights on human motor control strategies (Dipietro et al., 2007; Cheung et al., 2009, 2012; Safavynia et al., 2011). As an example, in the cited works of Tropea et al. and Camardella et al., stroke survivors' synergies were compared to healthy subjects' ones to investigate whether the patterns similarity reflect patients' cerebrovascular injuries and consequent functional recovery (Tropea et al., 2013; Camardella et al., 2020b). In another work of Steele et al., the authors checked how the choice of muscles can influence the synergy analysis by computing the similarity of synergies among different sets (Steele et al., 2013). In all these works, physiological meaning of altered synergies was assessed through the comparison with reference ones (healthy or unaltered).

For better accuracy and sensitivity in force estimation, featuring reaching tasks with either the whole upper limb or the wrist, simultaneous and proportional myo-control studies typically include 8–14 muscles, mainly large accessible muscles for surface EMGs. In the previous studies mentioned above, the rationale behind the number of chosen muscles was mainly based on human bio-mechanics. Attaching several EMG electrodes to the subject may be feasible in an experimental setting, but not comfortable for the subject. The absence of time constraints also makes such a set-up acceptable in an experimental environment. In rehabilitation robotics applications, the use of a large muscle set is not practical both for the subject and the therapist given the limited amount of time and resources.

The hypothesis of this study is that, given a specific myo-control application, the number of muscles to record can be reduced to a minimum set, concurrently preserving the performance of motor activity prediction and the physiological meaning of synergies. This will lead to three main achievements: (1) the reduction of inputs in a myo-control strategy and, consequently, a lower control processing time (i.e., computational cost), (2) the reduction in the number of EMG electrodes to apply on the subject, improving the comfort and ease of the setup, and (3) a higher sensitivity to changes of synergies, which could lead to a more evident motor function evolution and, if applied, to an ongoing modification of rehabilitation therapies. Nevertheless, the reduction in the muscle set size may affect the prediction capabilities of linear/non-linear models, and information on motor coordination that synergies provide. This last aspect comes from the fact that non-negative matrix factorization, used for synergies extraction, operates on the minimization of the root mean square residuals, between the input matrix and the product of output matrices, without any constraint on how muscle activities will be arranged in synergies: this means that any modification to the input matrix leads to different output patterns whose information on motor coordination may be altered. Thus, the objective of this study is to demonstrate that a minimum set of muscles, called optimal set, can be found, and that this set preserves performance of force/torque prediction and motor coordination information contained in synergies. The optimality is evaluated through the comparison between optimal and initial (namely full set) sets with two indexes: the difference in the prediction error (e.g., root mean square error, RMSE) and the correlation of synergies.

To do so, isometric contractions of nine healthy subjects, toward four directions in the horizontal plane, have been used to train a linear EMG-to-force model, for each possible combination of muscles in a total of 15, decreasing the size of the muscle set at each iteration. The authors searched for the optimal muscle set based on the RMSE of the EMG-to-force estimation in global and subject-specific conditions. These conditions depict how much a certain muscle is relevant for both all subjects (global condition) and for a specific subject (subject-specific condition), relying on a RMSE-based score. The preservation of force prediction performance has been evaluated through non-parametric statistical tools, assessing the absence of differences on the RMSE, between optimal and full set groups in both conditions. After that, the authors extracted muscle synergies from optimal and full sets and compared them through the Pearson correlation coefficient: the role of muscle synergies in this study has the aim of confirming the consistency of motor patterns generated by the optimal set. Moreover, two types of synergies have been tested for this purpose, in order to understand if the extraction process may influence the consistency of optimal set synergistic patterns.

## 2. Materials and Methods

### 2.1. Participants

Nine healthy individuals (age 24.9±1.3 years, weight 73.4±14.0 kg and height 177.1±5.7 cm, all males) participated in the study. All subjects were self-reported right hand dominant and at the moment of the experiment had no neurological, muscular, and orthopedic impairments. The experiment was their first experience with a setup that included EMG recording sessions. All subjects gave an informed consent prior to the study. The study has been approved by the Joint Chinese University of Hong Kong—New Territories East Cluster Research Ethics Committee and conducted in accordance with Declaration of Helsinki.

### 2.2. Experimental Setup

**Figure 1:**
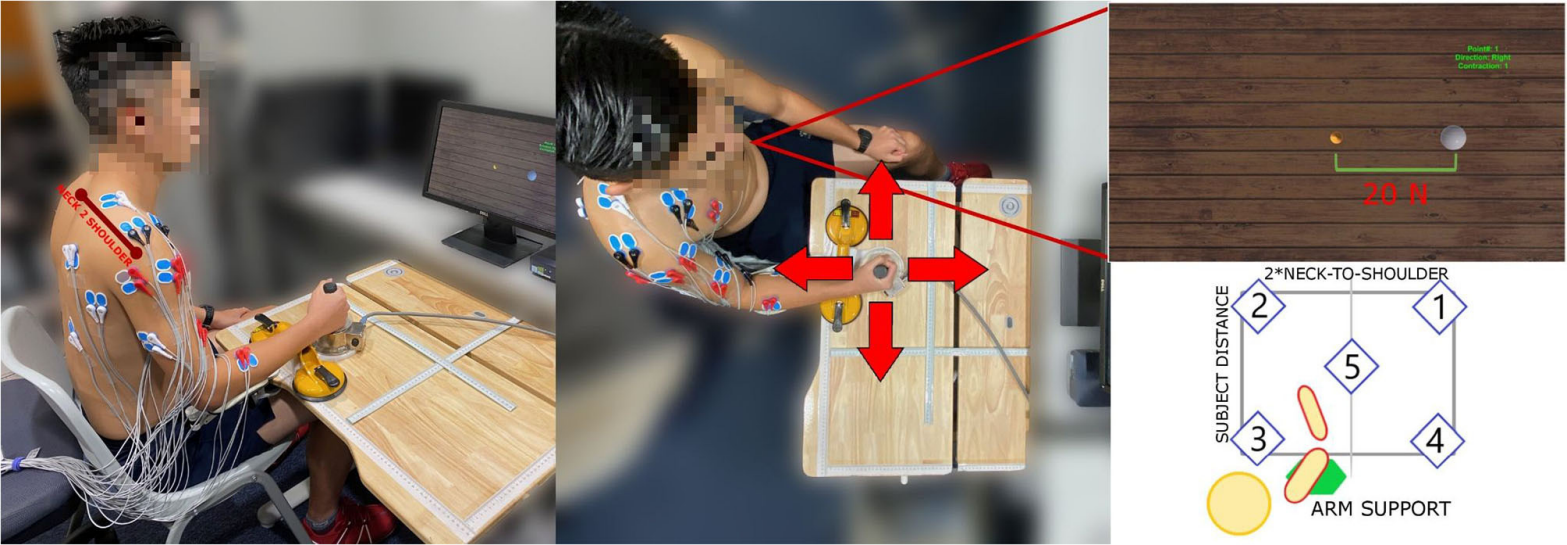
The experimental setup. Subject is seated in front of the workspace table, with the force sensor handle fixed in position. The bottom-right figure shows the five experimental sites. The subject shoulder joint is aligned with the central workspace position so the points are symmetrically placed with respect to the dominant arm. The workspace size is adapted on subject biometrics, having the width equal to the double of the neck-to-shoulder measure and the height such that the arm is never fully extended when reaching the furthest position.

The experimental setup was comprised of: (a) A 3D-printed cylindrical stationary joystick with ATI Gamma IP65 six-axis force/torque sensor (ATI Industrial Automation, Apex, NC, USA) with 65 N maximum load, that recorded forces generated at the hand and sampled at 125 Hz, fixed on a height-adjustable table, (b) an Ergorest elbow rest device for anti-gravity support (Ergorest Oy, Siilinjarvi, Finland) to lift tonic EMG signals resulted from sitting up with the arm raised to a table level, (c) a 16-channels surface EMG acquisition system with built-in band-pass and notch filters using two g.USBamp Biosignal Amplifiers (g.tec Medical Engineering GmbH, Austria), (d) a game environment shown on a monitor screen with a small golden ball representing the force generated cursor and a large white sphere representing the task target force (see Figure 1). The cylindrical stationary joystick was securely fixed in a certain position of the table. The central position (Position 5, in Figure 1) distance from the subject was calculated to be reachable with the subject's elbow at a 90 degree position. Surface EMG electrodes were placed after a thorough skin preparation based on the Surface Electromyography for the Non-Invasive Assessment of Muscles-European Community Project recommendations. Fifteen muscles from the dominant arm and torso were recorded for analyzing contralateral and ipsilateral contractions, as well as pushing and pulling actions, often related to reach-and-grasp movements. The full muscle set included: flexor digitorum (HAND FLEX), extensor digitorum (HAND EXT), biceps long head and short head (BI LO and BI SH), brachialis (BRACH), triceps lateral head and long head (TRI LAT and TRI LON), anterior deltoid (DELT A), medial deltoid (DELT M), posterior deltoid (DELT P), pectoralis major (PECT M), infraspinatus (INFRASP), upper trapezius (TRAP), latissimus dorsi (LAT DORSI), and teres major (TER MAJ). Ground electrodes were placed on the clavicle and the scapular acromion. Maximum voluntary contractions (MVC) for all muscles were observed at the beginning of the data collection for the EMG signals normalization. Each MVC was performed with a 1-min rest in between to avoid the effects of fatigue. EMG signals were acquired at a 1,200 Hz sampling frequency, as well as band-pass (5–500 Hz) and notch (50 Hz) filtered. The EMG acquisition PC was synchronized with the game environment and the PC that recorded the force/torque sensor using a User Datagram Protocol (UDP) connection between the two PCs.

### 2.3. Study Protocol

Subjects had to perform isometric contractions with the upper-limb in position, grabbing the handle in 5 sites of the horizontal workspace (see Figure 1). Subjects were seated on a stationary chair that was positioned to align the sternum with test positions 2 and 3, having the center of the shoulder joint approximately aligned with position 5. Subjects' neck-to-shoulder, arm and forearm measures were acquired to create a feasible workspace, symmetrical with respect to the dominant hand. Subjects grabbed the joystick, after placing their elbow on the anti-gravity support attached to an height-adjustable table. All subjects maintained their elbow at height with the help of the arm support and their distance from the workspace was computed using arm and forearm measures, in such a way that the furthest position was always reachable without the arm being in singularity. In each test position, subjects had to move the small golden ball cursor by generating force at the hand toward the target force (indicated by a large white sphere) in 4 different directions (forward, backward, right, and left). Each direction was repeated two times making a total of 8 trials. Subjects performed isometric muscle contractions to generate the force on the joystick. Target reach was deemed successful if the subjects could maintain the center of the ball cursor in the white sphere for 2 s. When subjects relaxed, meaning zero force input on the joystick, the small golden ball cursor returned to its original rest position. The start of the trial was indicated by the white sphere target appearing and the end was indicated by the white sphere disappearing. The white sphere target area is larger than the small golden ball cursor, indicating a tolerance of 5 N on the force target. A spring model *P*_*C*_ = K*F_*J*_ has been used to compute the position of the cursor (*P*_*C*_) using the measurements from isometric force exerted on the joystick (*F*_*J*_) with K as the elastic constant of the virtual spring (Berger and d'Avella, 2014).

### 2.4. Signal Processing and Dataset Splitting

Before training and testing the model, raw EMG signals were rectified and filtered using a 4 Hz 2nd order Butterworth low-pass filter and then normalized using the MVC. Then, the processed EMG dataset was split in training and test sets. Since each subject performed two contractions for each direction (see section 2.3), in every test of this study, one was randomly selected to be part of the training set and the other one to be part of the test set. For each subject, a datetime-dependent seed was used to determine a random sequence of numbers, as wide as the total number of combinations of muscle sets. Each value of this sequence uniquely selected a specific combination of contractions, to be used in the training set, taken as a 4-digits binary combination (one digit for each direction): if 0 the first contraction was used, otherwise the second one was included. The complementary sequence was used for building the test set. The training set was used for training the linear regressor only (see section 2.5) while the test set was used to build force estimations and extract performance indexes (see section 2.6.1): these indexes were used for the selection of optimal sets (sections 2.6.2 and 2.6.3) and the statistical analysis (section 2.6.4).

### 2.5. Model Building and Force Estimation

Muscle activations were always lower than the MVC value and in each workspace position the arm pose was fixed. Following this, the relation between the force exerted at the hand and the aforementioned filtered and normalized EMG signals, measured from elbow and shoulder muscles, was approximately linear (Buchanan et al., 2004; Berger and d'Avella, 2014; Buongiorno et al., 2018). The multi-variate linear regression algorithm (MVLR), that assumes a direct relation between muscle activations and hand-force exertion, has been used as the EMG-to-force model, currently being the most used in the state of art. Thus, the evaluation of performance of each muscle set was achieved comparing the force measurements with the estimations that resulted from the linear model, trained as following:


(1) $$\begin{array}{r} H=\underset{H\in {\mathbb{R}}^{n}}{\mathrm{argmin}}∥Hm_{t}\left(t\right)-F_{t}\left(t\right){∥}_{2} \end{array}$$

where *m*_*t*_ is the training EMG data matrix (*M* × *N* where *N* is the number of samples) and *F*_*t*_ is the training forces data matrix (2 × *N* matrix): thus, *H* will be a 2 × *M* matrix. In the case of a full muscle set *M* was equal to 15, otherwise it was equal to the chosen optimal muscles number. The subscript “t” in Equation (1) means that training set signals only should be used as the regressor training process relies on the training set only. Under the linear force-EMG relationship, the force estimation can be computed using the following equation:


(2) $$\begin{array}{r} F_{est}\left(t\right)=H\cdot m\left(t\right), \end{array}$$

where *F*_*est*_(*t*) is the estimated 2-dimensional force, *m*(*t*) is the processed test EMG signal and *H* is the aforementioned regression matrix. Force prediction (i.e., *F*_*est*_) could be potentially computed for both training and test sets, for example for RMSE computation on the training set, if needed.

### 2.6. Data Analysis

#### 2.6.1. Performance Indexes

Two different indexes have been used for assessing the force estimation performance of each method in each condition: Root Mean Square Error and Coefficient of Determination.

- Root Mean Square Error (*RMSE*)
  This index is used to measure the difference between the measured and the estimated forces and it is calculated as follows:
(3) $$\begin{array}{r} RMSE=\frac{\sqrt{\frac{\sum_{i=1}^{N}\left(x_{x,i}^{2}-{\hat{x}}_{x,i}^{2}\right)}{N}}+\sqrt{\frac{\sum_{i=1}^{N}\left(x_{y,i}^{2}-{\hat{x}}_{y,i}^{2}\right)}{N}}}{2} \end{array}$$
  The *x*_*x, i*_ and *x*_*y, i*_ are the x and y components of the *x*_*i*_ 2D measured force sample, respectively. The ${\hat{x}}_{x,i}$ and ${\hat{x}}_{y,i}$ are the x and y components of the ${\hat{x}}_{i}$ 2D estimated force sample, respectively. N stands for the number of samples. The lower the value of the RMSE the closer the estimated force matches the measured force signal amplitude.
- The Coefficient of Determination (*R*^2^)
  The *R*^2^ index is used to highlight a signal total variation explained by the estimates. The *R*^2^ is computed as follows:
(4) $$\begin{array}{r} R^{2}=1-\frac{SS_{res}}{SS_{tot}}=1-\frac{\sum_{i=1}^{N}{\left(x_{i}-{\hat{x}}_{i}\right)}^{2}}{\sum_{i=1}^{N}{\left(x_{i}-{\bar{x}}_{i}\right)}^{2}}, \end{array}$$
  where *x*_*i*_ is the original signal and ${\hat{x}}_{i}$ is the estimated output sample. *N* stands for the number of samples. The index ranges from minus infinite to 1 (equal to 1 in case of a perfect estimation with an error equal to zero).

#### 2.6.2. Muscle Scores

It was necessary to evaluate the performance of all muscle set combinations given the initial full set. To do so a loop was implemented defining the muscle set size at each iteration and then cycling on all combinations of muscles: the number of muscles was iteratively decreased from 15 to a minimum set of 4. For each muscle set a linear model was trained (see section 2.5) and tested on a different set of contractions (see section 2.4). Then a force estimation was built, according to 2, using test set signals, and compared to the measured ones through the RMSE index. Each time a performance index was computed, the muscles involved got a score equal to the RMSE value (averaged on the two force components) and summed to the previous score value. At the end of this loop, muscles with the lowest score were the most significant for that subject as they were always included in the sets that achieved the lowest estimation RMSE. A “ranking” of muscles was created, following the ascending order of RMSE scores and assigning to each muscle a rank equal to the ranking position: the first muscle in the ranking got a rank equal to 1, the second one a rank equal to 2 etc. This loop was repeated for all the participants. At the end of this analysis, all the muscles scores and ranks, for all the participants, were available to be used. A step-by-step procedure is shown below in the Algorithm 1.

**Table:** Algorithm 1:

Set of operations for evaluating muscle scores and ranks (i.e., importance) for a given subject. A “for” loop was implemented decreasing the i-th muscle set size from 15 to a minimum set of 4 at each iteration and then cycling on all combinations of muscles of the i-th set size. First operations concern the signal processing: envelope() refers to the low-pass filter used to extract the signal envelope and linreg() refers to the linear regressor training function. comb() function selects the j-th combination of muscles with i elements, while datasplit() randomly divides the dataset into training and test set. rmse() computes the RMSE index from measured and estimated forces. sort() function extracts the sorting indexes of the input, following either the ascending or descending option: for example, with the ascending option, if the forth muscle got the lowest score, the first element of ranks was equal to 4. This algorithm is repeated for all the participants. Ranks variable refers to the subject-specific rank values.
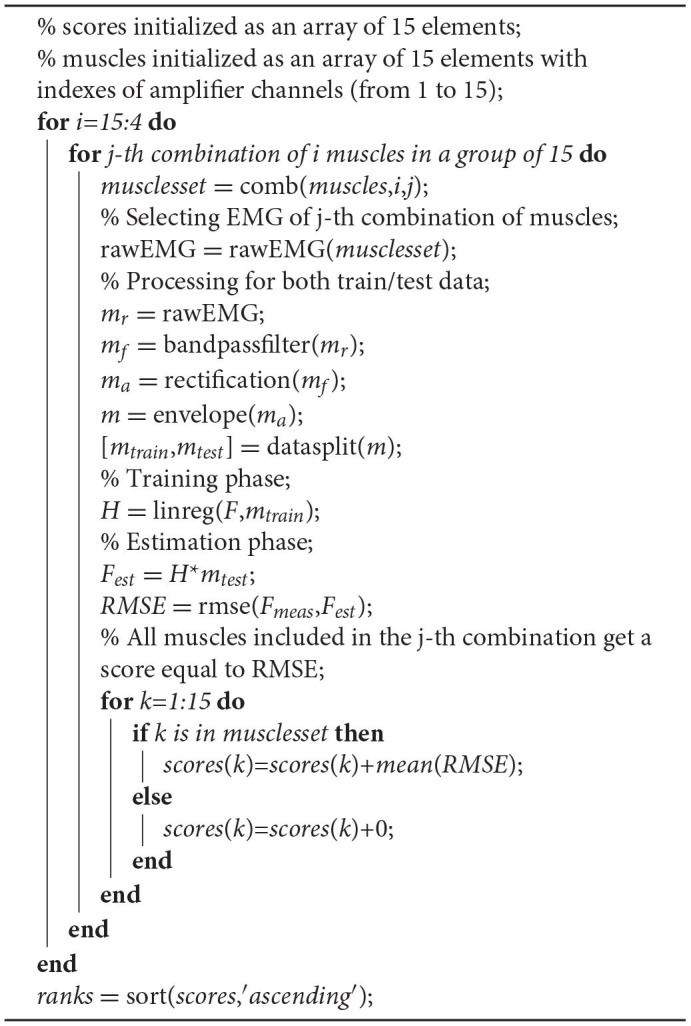

#### 2.6.3. Selection of Optimal Sets

Once the ranks of muscles have been obtained for all the subjects, actual performance of muscle sets could be computed without considering all of their combinations. This next step is divided in two analyses: a subject-specific one and a global one. In this step, RMSE of force estimation is computed iterating on all combinations of train/test datasets and on the number of muscles from 15 (full) to 4 (minimum).

- Subject-specific: At each iteration, the muscles to be used in the set were chosen by first discarding muscles with the highest rank (i.e., highest RMSE) in the subject-specific ranking.
- Global: This analysis differed from the previous one by the muscles choice criterion. In this case, a single global ranking was created summing all subject-specific rankings. The same loop as the previous point (i.e., subject-specific) was then performed: this time, at each iteration, the muscles to be used in the set were chosen by first discarding muscles with the highest rank (i.e., the highest RMSE) in the global ranking.

Eventually, from 15 to 4 muscles, a complete picture of the force estimation performance, for all the participants in both conditions, was depicted. The minimum number of muscles that showed a comparable amount of error (in terms of mean and standard deviation of the RMSE) w.r.t the full set, was chosen to be the optimal set. This optimal set then underwent both synergies computation process and statistical analysis, to assess its usability in a myo-control application.

#### 2.6.4. Statistics

The aim of the statistical study was to assess the expected similarity between optimal and full muscle sets force estimation performance. Thus, after choosing the optimal set to be analyzed, an ANOVA 1-way test was performed on the two populations: the acceptance of the null hypothesis would have confirmed the absence of significant differences among the two groups. Before launching the ANOVA test, Shapiro-Wilk normality test and χ^2^ homogeneity of variance test were performed on the two datasets, fulfilling parametric-test requirements. The analysis was repeated for both subject-specific and global optimal sets, compared to the full set.

### 2.7. Synergies

#### 2.7.1. Synergies Extraction

As done in previous works, muscle synergies could be extracted from electromyographical signals using the Non-Negative Matrix Factorization (NNMF) algorithm (Lee and Seung, 1999). This has been often chosen to separate the fundamental components from the input, assuming that negative muscle activations could not be physiologically obtained. In previous works, the NNMF identified the correct muscle synergies and activation coefficients in simulated data, combined with their consistency when applied to physiological data sets. Also NNMF was able to reconstruct the original signal in a similar way with reference to other more complex algorithms (Tresch et al., 2006). As noted in the literature four synergies were enough to describe electromyographical signals total variance in planar reaching tasks (Roh et al., 2012; Steele et al., 2013; Berger and d'Avella, 2014) with both isometric and dynamic setups. Thus, always four were the synergies extracted from optimal and full sets. As specified in the introduction the only role of synergies was to assess the coherence of optimal set patterns. NNMF was launched using the alternating least squares algorithm option in MATLAB “nnmf” function, with a maximum of 100 iterations and a tolerance of 10^−7^. According to the NNMF algorithm, muscle synergies can be computed as following:


(5) $$\begin{array}{r} m=W\cdot c+e_{m}, \end{array}$$

where *m* is the input signal (*M* × *N* matrix, being *M* the number of muscles and *N* the number of samples), *W* is the synergy matrix (*M* × *s* matrix, being *s* the number of synergies), *c* is the synergy activations matrix (*s* × *N* matrix) and *e*_*m*_ is the muscle activation factorization residuals, dimensionally equal to the input. Having multiple upper limb poses in the experimental protocol, muscle synergies could be extracted either in each of them or merging the information of all poses in a single synergies set. The latter has shown to be the most feasible one in synergy-based myo-control contexts, and it can be obtained in many ways (Buongiorno et al., 2017, 2019, 2020). “*Pose-Shared”* and “*Pose-Related,”* have been extensively detailed in a previous work and compared in this study, since they showed different adherence to the input dataset (Camardella et al., 2019). Briefly, the “*Pose-Shared”* synergies (herein called *W*_*g*_) are extracted running the NNMF once on a single EMG dataset using the Equation (5), in which *m* has been taken as the result of the union of the signals of each upper limb pose (*m* = [*m*_1_∪*m*_2_∪*m*_3_∪*m*_4_∪*m*_5_]). Instead, the “*Pose-Related”* synergies resulted from clustering P (i.e., number of poses) synergies sets, using the k-means algorithm, independently extracted from each arm pose and ordered with a minimum cosine distance criterion:


(6) $$\begin{array}{r} W_{c}\left(i\right)=kmeans\left(\overset{P}{\underset{p=1}{⋃}}W_{p}\left(i\right)\right),i\in \left[1,s\right], \end{array}$$

where the output *W*_*c*_(*i*) corresponds to the i-th element of the “*Pose-Related”* synergies matrix *W*_*c*_, as the i-th centroid of the clustered synergy vectors. *W*_*p*_(*i*) is the i-th synergy of *W*_*p*_ synergies matrix extracted in the point *p* (i.e., an upper limb pose): *P*, thus, is the total number of points (i.e., 5). The “*Pose-Related”* synergies matrix, *W*_*c*_, can be computed as the union of all the *s* centroids, given *s* the number of synergies:


(7) $$\begin{array}{r} W_{c}=\overset{s}{\underset{i=1}{⋃}}W_{c}\left(i\right). \end{array}$$

#### 2.7.2. Synergy-Based Model Building and Force Estimation

The synergy-based models followed the same concepts showed in section 2.5, thus, they were based on a linear relationship between the force at the hand and muscle activations, mapped in the synergy space using the synergies matrix (i.e., *W*). EMG signals were processed in the same way as done in section 2.5. The model was trained as following:


(8) $$\begin{array}{r} \begin{array}{c} H=\underset{H\in {\mathbb{R}}^{n}}{\mathrm{argmin}}∥Hc_{t}\left(t\right)-F_{t}\left(t\right){∥}_{2},\\ c_{t}\left(t\right)=W^{+}m_{t}\left(t\right) \end{array} \end{array}$$

where *c*_*t*_ is the training synergy activations data matrix (*s* × *N* where *s* is the synergies number and *N* is the number of samples), *m*_*t*_ is the training EMG data matrix (*M* × *N* with *M* defining the number of muscles), *W*^+^ is the pseudo-inverse of the *W* matrix (*s* × *M* matrix), and *F*_*t*_ is the training force data matrix (2 × *N* matrix): thus, *H* is a 2 × *s* matrix. In the case of a full muscle set, *M* was equal to 15, otherwise it was equal to the chosen optimal muscle number. Moreover, the synergy matrix *W* was chosen as *W*_*g*_ when using “*Pose-Shared”* synergies (PSS model) and *W*_*c*_ when using “*Pose-Related”* synergies (PRS model). Also in this case, the subscript “t” in Equation (8) means that training set signals only should be used as the regressor training process relies on the training set only. Finally, the force estimation could be obtained using the following formula:


(9) $$\begin{array}{r} F_{est}\left(t\right)=H\cdot c\left(t\right), \end{array}$$

where *F*_*est*_(*t*) is the estimated 2-dimensional force, *c*(*t*) is the synergy activation signal (built using either training or test EMG signals), and *H* is the aforementioned regression matrix computed using 8. Also in this case, force prediction (i.e., *F*_*est*_) could be potentially computed for both training and test sets, for example for RMSE computation on the training set, if needed.

#### 2.7.3. Synergy Similarities

After accomplishing the analysis described in section 2.6.3, synergies extracted from the optimal and the full muscle sets have been compared for evaluating the consistency of each synergy. The Pearson correlation coefficient (r) was used for evaluating the total similarity, as the average of coefficients of each optimal-full set pair of synergies. Given two sets (from optimal and full muscle sets) with four synergies each, 16 different correlation coefficients were computed. The highest calculated coefficient identified the best match between synergy pairs. After each calculation, the previously chosen synergies from both sets were excluded to avoid double-counting. In each synergy, only the muscular contributions from the same muscles of both sets were used. As a result, 4 correlation values were obtained, indicating the best matching synergies among the extracted ones. This process was repeated for both global and subject-specific sets.

## 3. Results

**Figure 2:**
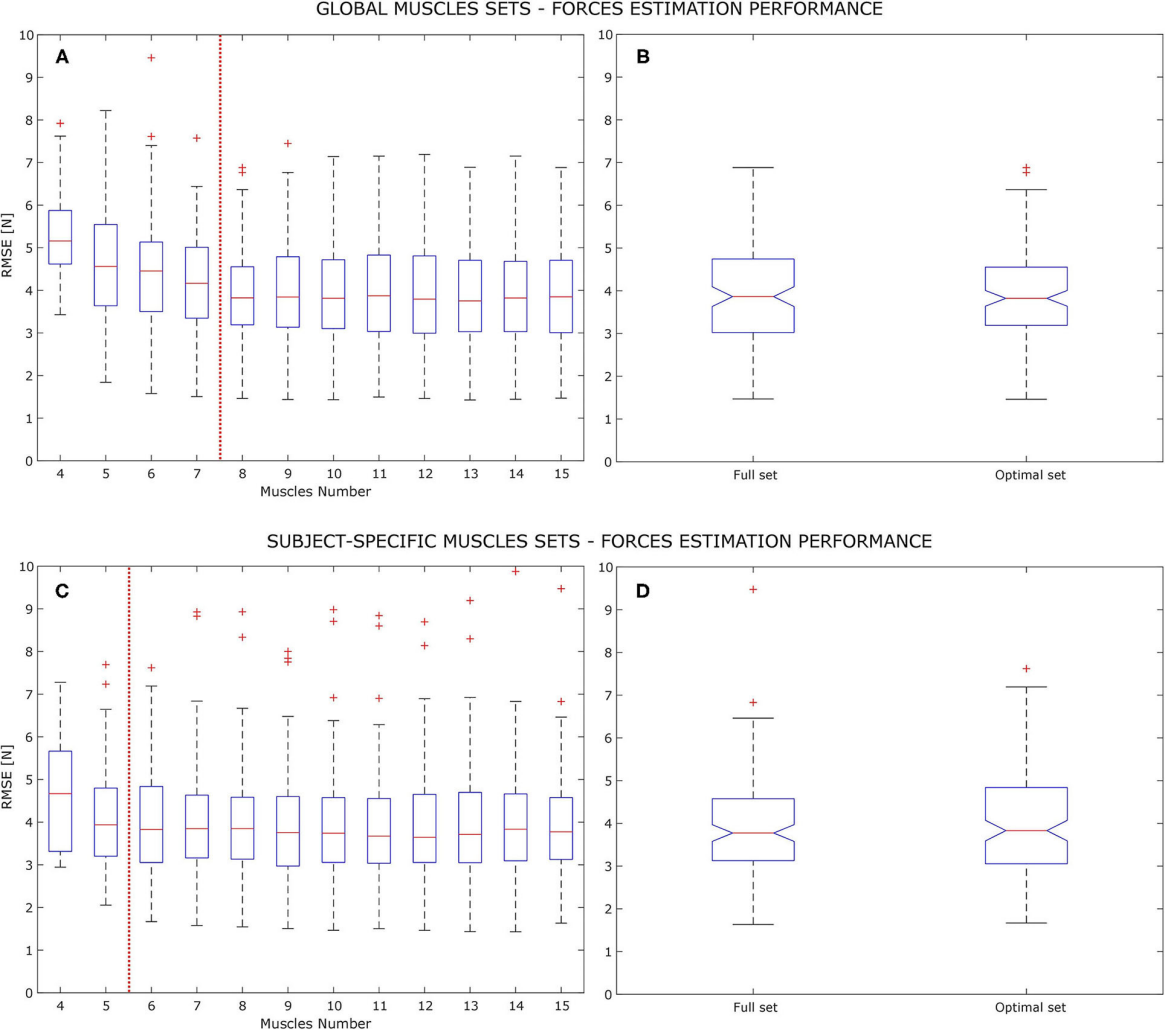
Force estimation RMSE, average of the x and y component and on the analyzed models (MVLR, PSS, and PRS), on the signals test set. Panels **(A,B)** are related to the global optimal set analysis while panels **(C,D)** are related to the subject-specific analysis, with increasing muscle set size. Figures on the left show all the RMSE box-plots, averaging all subjects performances on all the upper limb poses. Each box-plot show the errors quartiles with the horizontal red line representing the group median value. The red vertical dashed line show the stop criterion of the optimal muscle set search, indicating an increasing of either the median value or the total variance (indicated by the whiskers). Figures on the right show the 1-way ANOVA results for the 8 muscles global optimal set and the 6 muscles subject-specific optimal set, respectively, on panels **(B,C)**.

The main outcome of this study is that an optimal muscles set for the analyzed myo-control application does exist. Referring to Figure 2, global and subject-specific conditions are shown, regarding the RMSE performance analysis. In the global condition (Figure 2A), it was observed the RMSE value not to suffer from strong variations, both in variance and mean when varying the number of muscles from the full set (i.e., 15 muscles) to 8 muscles. Thus, the 8-muscles set has been taken as the global optimal muscle set, reporting 3.93±1.10 N as its value. Both full and optimal set observations passed the Shapiro-Wilk normality test. The chi-squared homogeneity of variance test was performed giving χ^2^ = 114.74 and *p* = 0.231 as values, confirming the homogeneity of variance null hypothesis (1.40 N full set variance, 1.20 N optimal set variance). According to 1-way ANOVA results, there was not a statistical difference between optimal and full muscle set performances giving *F*_(1, 13)_ = 0.02 with *p* = 0.886 as results. Figure 2C instead shows the variation in force estimation performance in the subject-specific condition. In this case the RMSE does not highlight any significant variation until it reaches 6 muscles. Reducing the muscles number from 6 to 4 brings to an increase of the RMSE mean by 0.10 N at 5 muscles and by 0.73 N at 4 muscles. It has to be noted that reducing the muscle set from 6 to 5 elements increases the total variance, bringing the minimum RMSE value from 1.67 to 2.05 N. According to this, the 6-muscle set has been taken as the subject-specific optimal muscle set, reporting 3.99±1.11 N as the force estimation RMSE. Also in this case full and optimal set observations passed the Shapiro-Wilk normality test and the chi-squared homogeneity of variance test with χ^2^ = 121.34 and *p* = 0.452 (1.40 N full set variance, 1.24 N optimal set variance). The 1-way ANOVA test did not show any statistical difference between optimal and full sets, with *F*_(1, 13)_ = 0.35 and *p* = 0.561.

**Figure 3:**
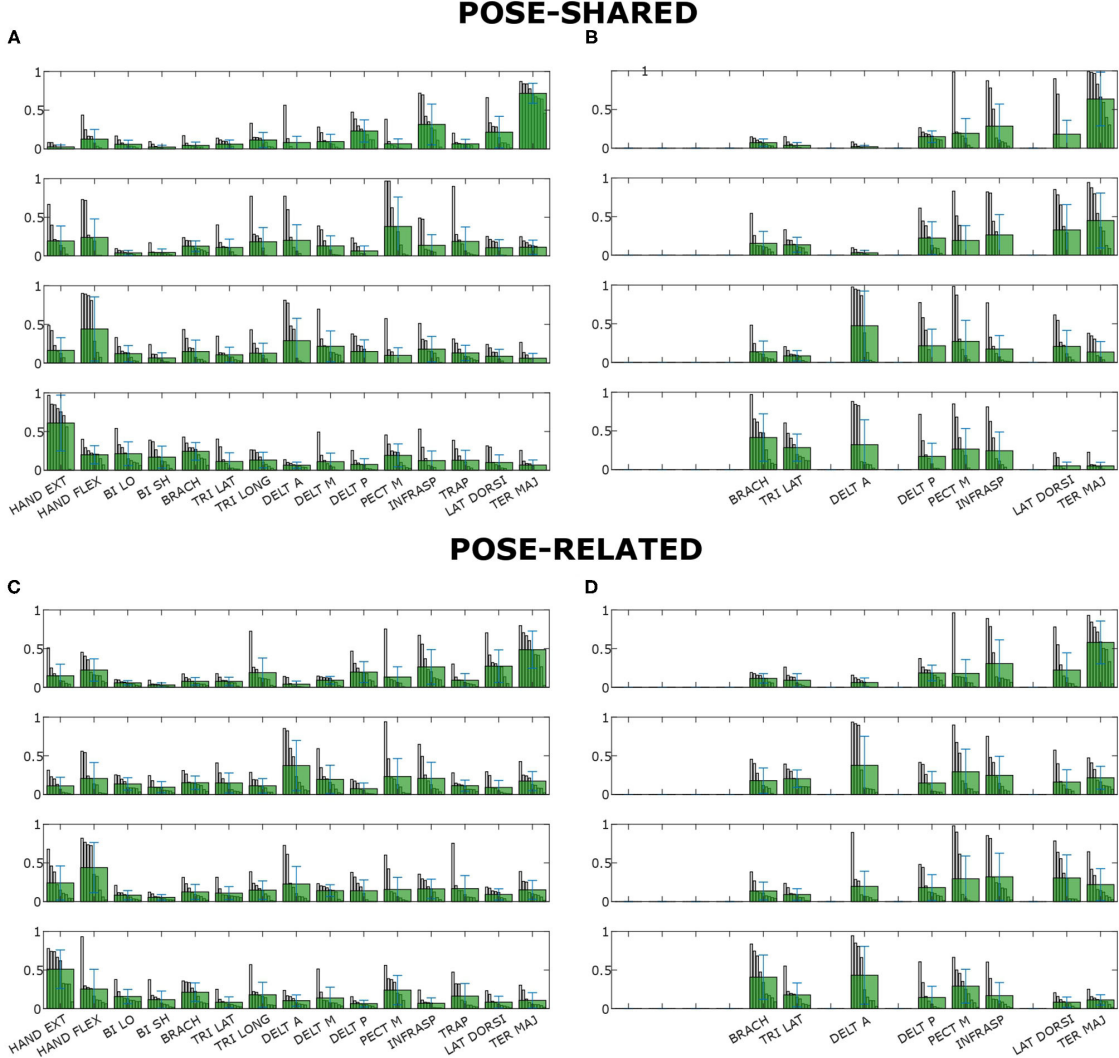
Synergies composition for “*Pose-Shared”* and “*Pose-Related”* extraction methods for both full and global optimal sets. Subject-specific synergy patterns could not be compared since subjects' muscle scores are generally different leading to different compositions of sets. Figures on the left **(A,B)** show the full set synergies composition, figures on the right **(C,D)** the global optimal sets composition. Each row (i.e., synergy) contains all muscle contributions, showing all the subjects' coefficients of that muscle in that synergy, previously ordered, as a gray bar. The light green bars represent the mean contribution values. Whiskers represent their total variance.

**Figure 4:**
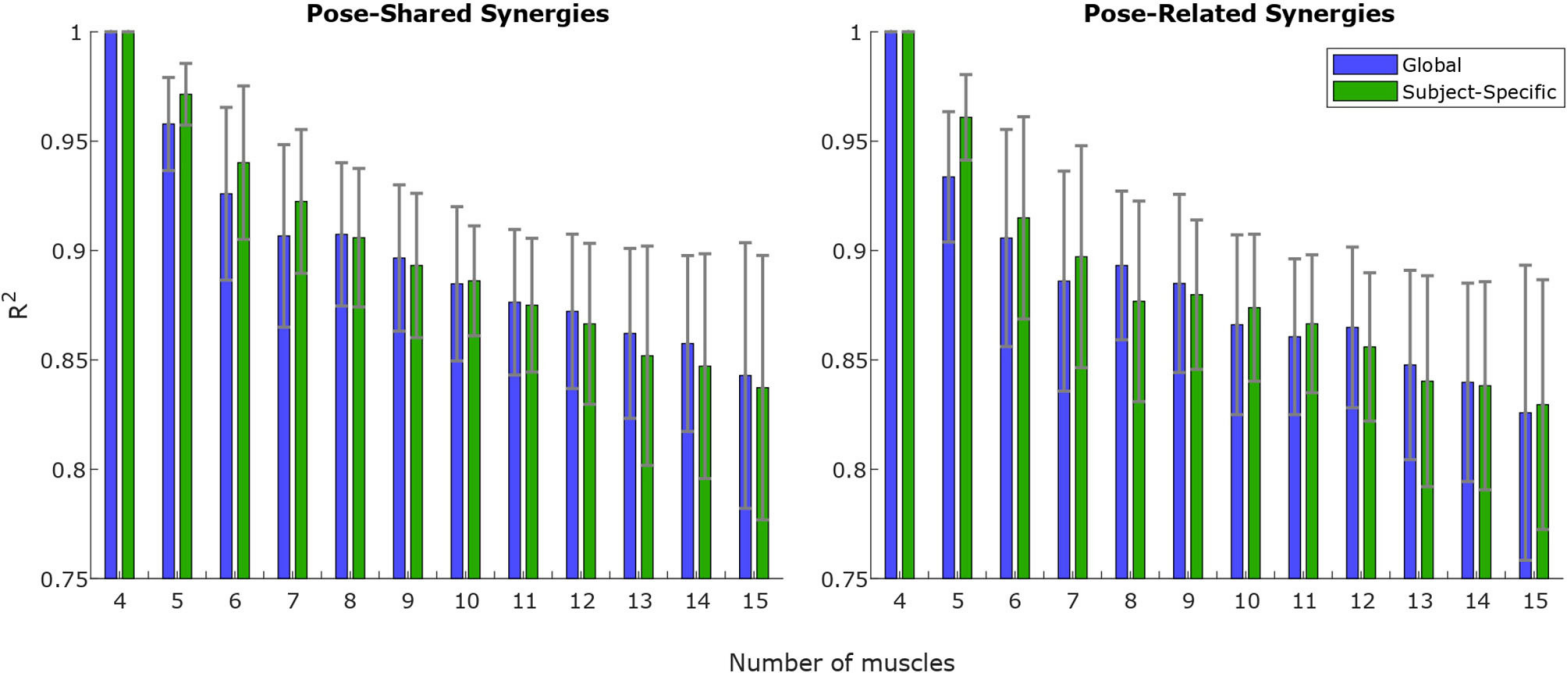
*R*^2^ ongoing mean and standard deviation values for increasing muscle set size, on “*Pose-Shared”* and “*Pose-Related”* synergies. Each value is computed by reconstructing training EMG signals using NMF outputs (see Equation 5) without residuals. The *R*^2^-value is computed on EMG signals merged from all the experimental workspace sites. The smaller the muscle size is, the higher the difference is in the EMG variance explained between Global and Subject-Specific conditions: for a lower number of muscles the choice of the muscle set, for each subject, that achieves the lowest estimation error, determines a higher reconstruction rate in terms of *R*^2^. This behavior is more evident when the number of muscles is lower than 8.

Concerning synergy similarities, both global and subject-specific optimal sets were analyzed, comparing synergies extracted from these sets to the full set ones. In the former case, synergies showed a mean correlation value of *r*_(6)_ = 0.74, *p* = 0.035 for the “*Pose-Shared”* synergies and *r*_(6)_ = 0.71, *p* = 0.048 for the “*Pose-Related.”* In the latter case, a mean correlation value of *r*_(4)_ = 0.78, *p* = 0.067 with “*Pose-Shared”* synergies and *r*_(4)_ = 0.71, *p* = 0.113 for “*Pose-Related”* synergies was found. Figure 3 depicts the comparison of synergistic patterns between full and global optimal muscle sets. All the values represent the average across subjects. *R*^2^ values for “*Pose-Shared”* synergies generally advantaged the subject-specific condition in almost all cases (see Figure 4). Excluding the 4-muscles case, which matched the number of synergies, synergies that exploited the global sets achieved an EMG reconstruction rate ranging from a minimum of 0.878±0.028 to a maximum of 0.972±0.016. In the subject-specific case the *R*^2^ scored 0.880±0.031 with 15 muscles up to 0.982±0.009 with 5 muscles.

The last result regards linear regression coefficients that constitute the actual link between muscle/synergy activations and the amplitude and direction of the generated force vectors. With the aforementioned 8-muscles global optimal muscle set, the mean Pearson correlations value of regression coefficients, between the full muscle set and the optimal set, scored *r*_(6)_ = 0.93, *p* < 0.001 for MVLR, *r*_(6)_ = 0.88, *p* = 0.004 for “*Pose-Shared”* and *r*_(6)_ = 0.89, *p* = 0.003 for “*Pose-Related.”* Instead, exploiting the 6-muscles subject-specific optimal muscle set, the mean correlation of regression coefficients was *r*_(4)_ = 0.87, *p* = 0.024 for MVLR, *r*_(4)_ = 0.85, *p* = 0.032 for “*Pose-Shared”* and *r*_(4)_ = 0.89, *p* = 0.021 for “*Pose-Related.”*

## 4. Discussions

The search for an optimal muscle set, in the planar myo-control application, gave a positive answer. The analysis on a pool of 9 healthy subjects led to two different optimal muscle sets, depending on the selected muscles choice criteria. If a subject-specific optimal muscle set was chosen, muscles could be reduced up to a minimum of 6, resulting in a loss of correlation significance with full set synergies while keeping similar estimation performance, and significant muscles-to-force and synergies-to-force coefficients coherence. This comes from the statistical analysis results from which no statistical significance was found between 6-muscles and 15-muscles groups (*p* = 0.350), regarding force estimation RMSE, while force fields showed a statistically significant correlation (*p* = 0.024 for MVLR, *p* = 0.032 for Pose-Shared, and *p* = 0.021 for Pose-Related). The global optimal muscle set, i.e., 8 muscles shared across all the subjects, revealed to be a reliable subset on all analyzed indexes. This means having a similar force estimation error (no statistically significant differences between groups with *p* = 0.886) and significant synergy similarities with respect to the full set ones (Pearson correlation between synergies scoring *p* = 0.035 for “*Pose-Shared”* and *p* = 0.048 for “*Pose-Related”*). Also a statistical significance on the muscle-to-force and synergy-to-force mapping coefficients was found, enforcing the coherence of the optimal set composition. The global set, thus, counted two more muscles with respect to the subject-specific one but synergy similarities were comparable, reaching a statistical significance. Nevertheless, the force estimation performance given by the two optimal sets were comparable both in mean and standard deviation. This result suggests that although a subject-specific optimal set is functional in estimating the force in a certain application, synergies change their composition (i.e., the contribution of each muscle) when lowering the number of muscles under a certain threshold. In any case the global optimal set does not seem an obstacle to a fully working myo-control application, rather reducing the computational cost of the overall force estimation process when synergies are not used.

In the *R*^2^ graph shown in Figure 4, global and subject-specific optimal set synergies generally explain the original signal total variance in a comparable way, for both “*Pose-Shared”* and “*Pose-Related,”* until the 8-muscles set is reached. Below this threshold, as expected, synergies in the subject-specific set achieve a higher *R*^2^ value, since every subject could exploit a slightly different movement strategy that led to optimal sets that are different from the most shared one. This aspect leads to important implications in the rehabilitation context if synergies are used as an assessment tool. Without exploiting an optimal set, synergies already have shown to be important markers for detecting cortical damages or new skills acquisitions (Safavynia et al., 2011; Cheung et al., 2012; Tropea et al., 2013). The optimal muscle set, specifically selected for a stroke individual, could better highlight abrupt variations in synergy correlation with respect to initial patterns, after rehabilitation, meaning a change in movement strategy. As explained before, this is given by the computation of a similarity index of synergies that involves sub-computations on each muscle. Moreover, this specific set could better reflect how motor units in muscles are recruited for that specific subject, differently from the global set that may depict a generalized behavior. These suggestions will be deeply investigated in future studies, involving rehabilitation training in the analysis. Moreover, no strong differences have been found between “*Pose-Shared”* and “*Pose-Related”* synergies, with Shared synergies slightly outperforming the Related ones. In a previous work, with a larger workspace and a similar setup, the ability of “*Pose-Related”* synergies to reconstruct the initial EMG dataset was higher than “*Pose-Shared”* ones (Camardella et al., 2020a). This discrepancy in results may confirm the ability of “*Pose-Related”* synergies to better explain datasets that include limb poses that are very different, since synergies are extracted independently on each pose and clustered together.

The subject discomfort deriving from bulky setups and long-lasting preparations with a high number of electrodes could be alleviated thanks to reduced sets. Although subjects benefit from a subject-specific set, this would inevitably require at least one training session with a full set, to find his/her specific movement strategy and, thus, his/her optimal set. In the case of altered motor patterns, subjects would require multiple training sessions each time an abrupt drop in correlation with the initial patterns is detected. Moreover a global optimal set could be of difficult usage in this context, since stroke generally induces unpredictable alterations and a priori muscle set does not seem suitable (Dipietro et al., 2007; Roh et al., 2013, 2015; Camardella et al., 2020b). Nevertheless, a representative healthy global optimal set could be helpful as a comparison with physiological patterns.

Regarding differences between “*Pose-Shared”* and “*Pose-Related”* synergies, the former achieved a higher correlation on both subject-specific and global optimal sets. “*Pose-Related”* synergies keep a comparable correlation value between subject-specific and global optimal set. This suggests that “*Pose-Shared”* may suffer the changing of muscles in the set, instead of the “*Pose-Related”* ones that seemed more robust to those variations. In a previous work by Camardella et al. (2019), synergy-based myo-control strategies, with “*Pose-Shared”* and “*Pose-Related”* synergies, were compared on the test set on both RMSE of force estimation and EMG reconstruction performance. In this instance “*Pose-Related”* seemed to better trace upper limb features on different workspace sites, when using a full muscle set, as well as to better estimate the force at the hand. In another work (Camardella et al., 2020a), “*Pose-Related”* synergies were used in a synergy-based myo-control during an online virtual session, suggesting the feasibility of such method in estimating the hand force in real-time. In this study, the coherence of “*Pose-Shared”* and “*Pose-Related”* synergies have been investigated in the case of a reduced muscle set, under similar protocol and signal processing conditions. “*Pose-Shared”* synergies revealed to be more similar to the full set ones, with correlation values always higher than “*Pose-Related”* ones. Also, referring to Figure 4, they better reconstruct the original EMG signals, mostly having higher values of *R*^2^ with smaller muscle set size. This outcome may suggest that “*Pose-Related”* could be preferable in the case of a large muscle set, when directly involved in a synergy-based myo-control application trying to exploit the modular organization of the musculoskeletal system and projecting it onto the force task, rather than using it as an assessment tool.

**Figure 5:**
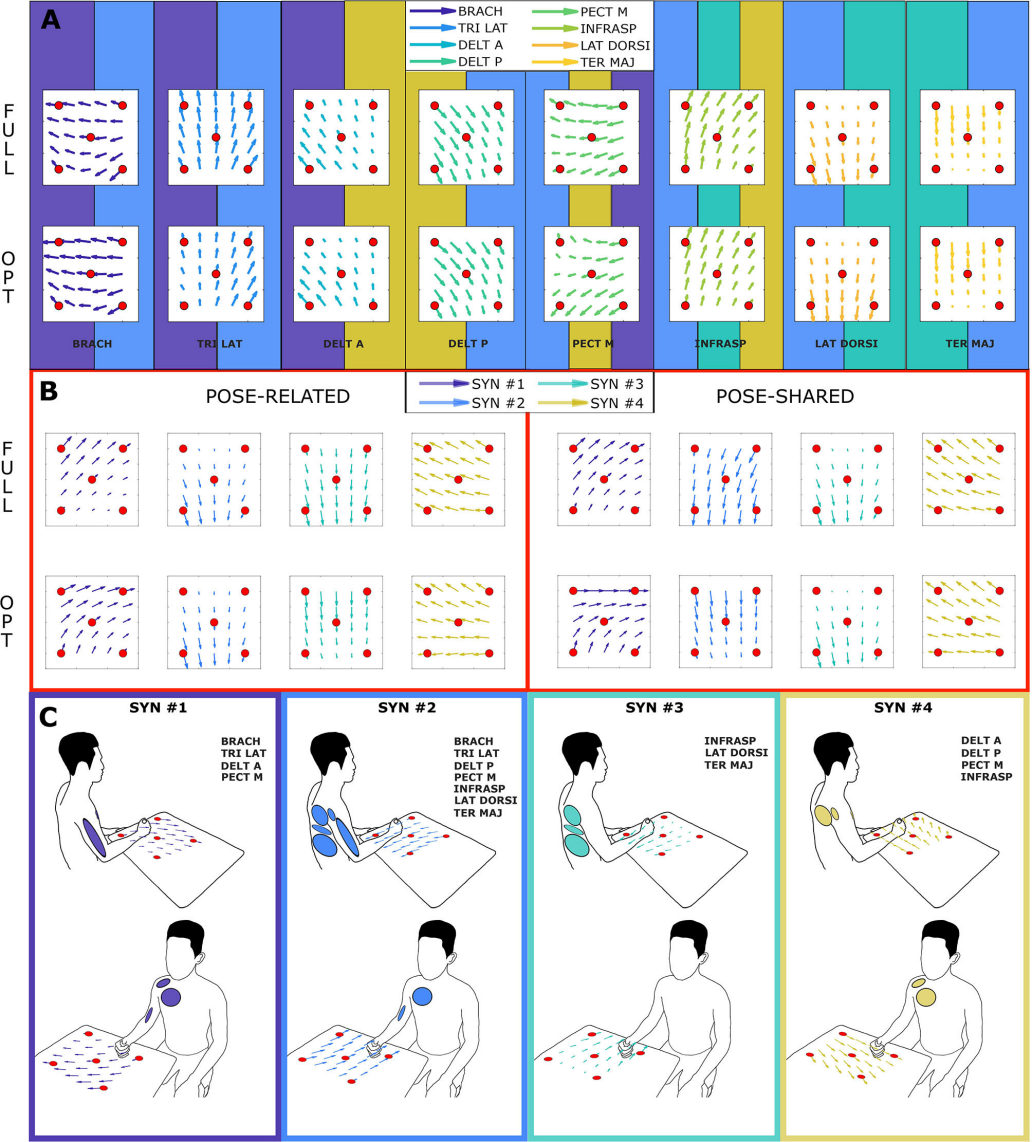
Amplitude and direction of pulling vectors of recorded muscles and extracted synergies (force fields), in the global optimal muscle set. Each force field interpolates the information of the columns of the H matrix (see sections 2.5, 2.7.1). Each red sphere represents the action of a muscle/synergy in one experimental point. Arrows are then interpolated in a 6 by 6 grid. The section **(A)** shows the force field of each muscle in the full and optimal set: muscle force fields are taken from columns of H matrix trained with Equation (1). The background tile of each muscle explains the synergy of influence: multiple tiles refer to as many synergies of the same color in the section **(B,C)**. The section **(B)** shows the force field of each synergy in the full and optimal set: synergy force fields are taken from columns of H matrix trained with Equation (9). The right panel illustrates the “*Pose-Related”* synergies force fields while the left one the “*Pose-Shared”* ones. The section **(C)** summarizes force fields and involved muscles, depicting the frame color and the muscle ellipses as the synergy color of the section **(B)**.

As proposed by Steele et al. (2013), it is possible to label as dominant those muscles that have the highest contribution in a specific synergy. Looking at the Figure 3, synergies do not show a big difference in the correspondence of dominant muscles between full and optimal sets, at a glance. In particular, as showed in Figure 5, it is important to associate muscle pulling vectors to synergies that group them and, eventually, to have a quick overview of how a specific synergy act in task-oriented movements such as reaching motions. When dominant muscles are included, a good variance accounted for can be achieved even with a low number of muscles, better than choosing them randomly. Although the task, from which the EMGs were recorded, was different, the optimal sets that have been found include most of the muscles showed to be important in the work of Steele et al. (2013) (TER MAJ, LAT DORSI, TRI LAT, and BRACH). Moreover the number of muscles found to be the most representative in the muscle set, corresponds to the minimal one found, which includes 5/6 muscles. According to this outcome, force estimation performance revealed to be a good muscles choice criterion, highlighting the link between synergies and force task. The global optimal set information showed in Figure 5 reveal the field of action of each muscle and synergy in the experimental workspace. Both the composition of synergies and their field of influence, in the global optimal set, trace common features already stated in the state-of-art. In a previous work by Cheung et al. (2009), synergies extracted from more than 12 muscles, during dynamic tasks, on a pool of seven out of eight total stroke subjects, revealed a strong similarity between affected arm and unaffected arm patterns. Among those patterns, there were synergies including co-activations of the brachialis and the triceps lateral head, the pectoralis major and the deltoid anterior, and the infraspinatus with the deltoid posterior and the teres major, as stated previously. Another work by Berger and d'Avella (2014), showed similar synergy compositions coming from eight healthy subjects, extracted from 13 muscles during isometric contractions, keeping a fixed pose of the upper limb. In all the cases, it is interesting to notice how some muscles (e.g., the pectoralis major or the infraspinatus) are not fully included in a single synergy but participate in multiple synergies (for example acting as rotator or stabilizer of the shoulder joint) with different contributions. Moreover, muscle and synergy force fields did not show strong differences between full and optimal sets, confirming that the global optimal set owned the most important features of the full set, concurrently bringing the aforementioned advantages. Also “*Pose-Shared”* and “*Pose-Related”* synergy differences did not seem remarkable, suggesting that both extraction methods may present consistent outcomes and be used wisely judging the right application field.

## 5. Conclusions

In this work, the existence and feasibility of an optimal muscle set to be used in a myo-control application has been investigated. An 8-muscles global optimal set, the best trade-off in terms of myo-control performance and the muscle set size, shared among the analyzed pool of subjects, has been found. The optimal set has shown no statistical differences in terms of force estimation performance and a high correlation with the initial (full muscle set) synergistic patterns. Also muscle and synergy force fields in the optimal set resulted to be coherent with the full counterpart. Tailoring the muscle choice to the specific subject, the optimal set could get to include up to 6 muscles, nevertheless loosing statistical similarity on synergies but retaining the ability to explain a higher variance of EMG signals, with respect to the global one, with the same number of muscles and synergies. A link between synergies and force task was identified, thus, dominant muscles that cover an important role in the chosen protocol can be found through the minimization of the force estimation error. Future studies will involve an actual usage of optimal sets in either a real-time myo-control application or an assessment tool for rehabilitation.

## Data Availability Statement

The raw data supporting the conclusions of this article will be made available by the authors, without undue reservation.

## Ethics Statement

The studies involving human participants were reviewed and approved by Joint Chinese University of Hong Kong—New Territories East Cluster Research Ethics Committee. The patients/participants provided their written informed consent to participate in this study.

## Author Contributions

CC, AF, and RT: conceptualization. CC, MJ, and RT: methodology. CC, MJ, and KT: software, validation, and visualization. CC: formal analysis. CC and MJ: investigation and writing—original draft preparation. MJ and KT: data curation. AF and RT: supervision. RT: project administration. AF: funding acquisition. All authors contributed to writing—review and editing and have read and agreed to the published version of the manuscript.

## Conflict of Interest

The authors declare that the research was conducted in the absence of any commercial or financial relationships that could be construed as a potential conflict of interest.

## Publisher's Note

All claims expressed in this article are solely those of the authors and do not necessarily represent those of their affiliated organizations, or those of the publisher, the editors and the reviewers. Any product that may be evaluated in this article, or claim that may be made by its manufacturer, is not guaranteed or endorsed by the publisher.
